# Late gadolinium enhancement CMR for detecting myocardial injury after cancer therapy: a real-world observational study

**DOI:** 10.3389/fcvm.2026.1787820

**Published:** 2026-05-01

**Authors:** Xi Liu, Yue Gao, Zhen Wang, Wei-Feng Yan, Rui Shi, Yuan Li, Ying-Shi Sun, Zhi-Gang Yang

**Affiliations:** 1 Department of Radiology, West China Hospital, Sichuan University, Chengdu, Sichuan, China; 2 Key Laboratory of Carcinogenesis and Translational Research (Ministry of Education/Beijing), Department of Radiology, Peking University Cancer Hospital & Institute, Beijing, China

**Keywords:** cancer therapy-related cardiotoxicity, cardiovascular magnetic resonance, late gadolinium enhancement, left ventricular, myocardial strain

## Abstract

**Background:**

Clinically, cardiotoxicity defined by reduced left ventricle ejection fraction (LVEF) in cancer therapy could miss the onset of tissue-level myocardial changes. In this study, we aimed to use late gadolinium enhancement (LGE) cardiac magnetic resonance (CMR) to evaluate myocardial injury in patients receiving cancer therapy, and to investigate the effect of LGE and other clinical factors on LV function.

**Methods:**

This study included 108 cancer patients and 60 healthy controls examined by CMR imaging. Patients were assigned to the LGE-negative (LGE−, *n* = 80) and LGE-positive groups (LGE+, *n* = 28). The LV functional parameters and myocardial strain parameters, were compared among the three subgroups. Associations between variables were evaluated via Pearson or Spearman correlation analyses. Further, the association between risk factors and LVEF was determined via multivariate linear regression analysis.

**Results:**

The LVEF and peak strain (PS) in all directions were significantly lower in the patients with LGE than in those without (all *p* < 0.05). There were moderate to high correlations between circumferential peak diastolic strain rate (PDSR), peak systolic strain rate (PSSR), PS, and LVEF in the patients with cancer (*r* = 0.54, *r* = −0.63, *r* = 0.82, respectively; *p* < 0.001). Multivariate linear regression analysis revealed independent associations between 1) N-terminal-pro B-type natriuretic peptide and radial, circumferential and longitudinal PS (*β* = −0.419, *β* = 0.407, *β* = 0.327, respectively; *p* < 0.001), 2) troponin T and circumferential PDSR (*β* = 0.342, *p* < 0.001) and between 3) the LGE extent (LGE%, 5SD) and circumferential PS (*β* = 0.297, *p* < 0.001).

**Conclusions:**

The presence of LGE was an important risk factor for LV dysfunction in patients receiving cancer therapy, and the circumferential strain reduction is the predominant mechanism of LV dysfunction.

## Background

Significant advancements in cancer therapeutics have led to improved long-term survival in patients with cancer, however, these therapeutic options are potentially cardiotoxic. Cancer survivors are at higher risk than the general population for developing cardiac disease, which is the leading cause of noncancer-related mortality in cancer survivors (1, 2). Timely and accurate identification of cancer therapy-related cardiotoxicity, which poses a formidable challenge for oncologists and cardiologists, is crucial to enable the targeted use of cardioprotective interventions and/or to introduce alternative treatment strategies (3).

Cardiac magnetic resonance (CMR) imaging has high spatial resolution and accuracy, and plays a very important role in the diagnosis and prognosis of various cardiomyopathies. Late gadolinium enhancement (LGE) imaging provides tissue characterization that enables detection of myocardial fibrosis and/or edema associated with cardiotoxic therapy (4, 5). Recently, myocardial strain analysis based on feature-tracking CMR has become a sensitive method for detecting subclinical myocardial dysfunction (6). Therefore, this study aimed to use LGE to characterize myocardial damage in a somewhat diverse population that included several cancer types and treatment regimens, and to investigate the effect of LGE and other clinical factors on left ventricular (LV) strain.

## Methods

### Study population

We conducted a retrospective study of patients with various cancer who were referred for clinical CMR for suspected cardiotoxicity (base on symptoms, cardiac biomarkers or echocardiography) between June 2018 and March 2025. The inclusion criteria were age >18 years and had received at least one antitumor treatment (including conventional chemotherapy, immunotherapy, targeted therapy, or radiotherapy). The exclusion criteria were pre-existing symptomatic heart failure (NYHA Class III or IV), a history of myocardial infarction, or chronic arrhythmia such as atrial fibrillation, incomplete medical records, or inadequate CMR studies. Besides, 2 cancer patients with coronary heart disease were excluded from the study cohort due to extensive ischemic LGE caused by silent myocardial infarction. We also included 60 age- and sex-matched normal controls who underwent CMR. The exclusion criteria were clinical evidence of cancer, cardiovascular disease, or other systemic disease or inadequate CMR studies. This retrospective study was approved by the Institutional Review Board, which waived the requirement for written informed consent.

### Clinical data extraction

Detailed clinical data were extracted from the electronic medical records. We collected the following data: demographic characteristics, vital signs, cancer treatments, previous cardiovascular history, cardiovascular risk factors, and cardiac biomarkers (at a similar time to the CMR) including serum N-terminal pro-B-type natriuretic peptide (NT-pro BNP) and troponin T. We then counted the number of cardiovascular disease risk factors (known coronary artery disease, hypertension, diabetes mellitus, smoking, or elevated cholesterol) to enable calculation of a cardiovascular disease score (range: 0–5).

### MR protocol

The CMR examinations were performed on 3T scanner (Tim Trio/Skyra, Siemens, Erlangen, Germany) with standard image acquisitions. The data included cine images acquired with a balanced steady-state free-precession sequence (TR: 2.9/3.4 ms; TE: 1.25/1.3 ms; flip angle: 50/40°; slice thickness: 8 mm; field of view: 319 × 249/339 × 284 mm^2^; matrix size 256 × 166/256 × 144), and LGE imaging using a T1-weighted inversion recovery turbo FLASH sequence (TR: 650/500 ms; TE: 1.43/1.24 ms, flip angle: 50/40°, slice thickness: 8 mm, field of view: 340 × 255/340 × 233 mm^2^, matrix size: 256 × 192/256 × 125) conducted for 10–15 min after intravenous administration of gadolinium-based contrast agent.

### Image analysis

All CMR imaging data were analyzed by an experienced radiologist (>8 years of experience in interpreting CMR) using commercially available software (cvi42 version 5.11.2, Circle Cardiovascular Imaging Inc., Calgary, Canada). The LV function parameters, including the end diastolic volume (EDV), end systolic volume (ESV), stroke volume (SV), and ejection fraction (EF), as described in a previous study were acquired (6). To analyze the LV myocardial strain, the short- and long-axis two-chamber and four-chamber slices of the cine images were loaded into the tissue-tracking module, as described in our previous study (7). The LV global strain parameters, including radial, circumferential, and longitudinal peak strain (PS), peak systolic strain rate (PSSR), and peak diastolic strain rate (PDSR) were estimated automatically.

**Figure 1:**
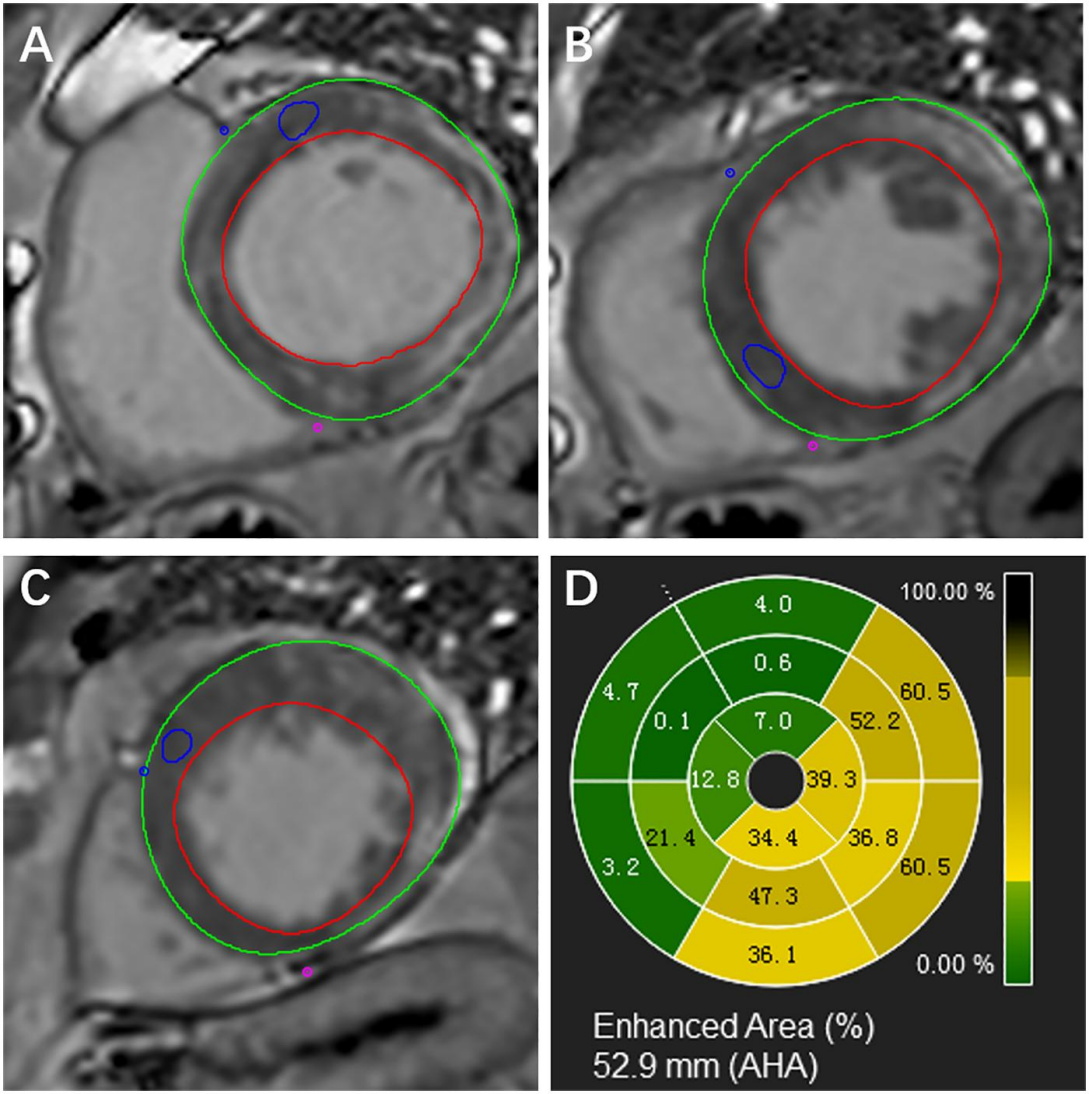
Representative LGE images of the basal **(A)**, middle **(B)**, apical **(C)** short-axis slices, and bull's eye image evaluated by semi-quantitatively **(D)** of LGE+ patient with cancer.

LGE was visually identified as positive (LGE+) if there were hyperintense regions within the myocardium in the short- or long-axis views. In addition, for LGE quantification, the extent of LGE based on the signal threshold analysis was performed using sequential short axis LGE images. LGE lesion was evaluated semi-quantitatively as above the five-fold standard deviation (5SD) of the remote reference myocardium that demonstrated the lowest signal intensity (Figure 1). The LGE extent was calculated as the ratio of the LGE lesion volume to the total LV myocardium volume.

### Statistical analysis

SPSS software (version 23.0), GraphPad Prism software (version 8.0.1), and R software (R studio version 1.4.1717) were used to perform the statistical analyses. Categorical variables are presented as numbers (percentages) and compared by performing Fisher's exact or Chi-squared tests. Continuous variables are presented as means ± standard deviations or as medians and interquartile ranges. Normality was evaluated by performing the Kolmogorov–Smirnov test, and the homogeneity of variance was assessed by performing Levene's test. Comparative analyses were repeated in subgroups after stratification of the study cohort into three categories (control, cancer patients without LGE, and cancer patients with LGE) by performing one-way analysis of variance, followed by Bonferroni *post hoc* correction or its nonparametric equivalent (Kruskal–Wallis test), as appropriate. Associations between variables were investigated by performing Pearson or Spearman correlation analyses. Furthermore, univariate factors of *p* < 0.1 were then included in the stepwise multivariate analysis. NT-pro BNP was log-transformed before inclusion in the regression analysis. Values of *p* < 0.05 were accepted as indicating statistical significance.

## Results

### Baseline characteristics

The final study cohort comprised 108 patients with cancer who received potentially cardiotoxic therapy and 60 healthy controls. On CMR images, LGE was identified in 28/108 (25.93%) of the patients with cancer. The patients with cancer without and with LGE were assigned to the LGE-negative (LGE−) group (*n* = 80) and LGE-positive (LGE+) group (*n* = 28). The baseline characteristics of the study population were presented in Table 1.

**Table 1:** Baseline characteristics of control individuals, cancer patients without or with LGE.

Characteristics	Normal *n* = 60	Patients (LGE−) *n* = 80	Patients (LGE+) *n* = 28
Age, y	55.00 (49.00, 63.00)	62.00 (50.00, 67.00)	60.00 (53.00, 71.00)
Male gender, *n* (%)	26 (43.33%)	41 (51.25%)	19 (67.85%)
BMI, kg/m^2^	23.52 ± 3.15	22.52 ± 3.75	23.42 ± 3.01
Heart rate, bpm	74.60 (66.91, 78.27)	79.96 (71.34, 94.09)	85.07 (75.14, 95.38)*
Diastolic BP (mmHg)	125.75 ± 15.42	1,119.57 ± 16.14	121.32 ± 18.76
Systolic BP (mmHg)	77.16 ± 9.98	77.51 ± 11.48	83.67 ± 14.85*
CV risk factors			
Smoking, *n* (%)	—	23 (28.75%)	8 (28.57%)
Hypertension, *n* (%)	—	20 (25.00%)	7 (25.00%)
Diabetes, *n* (%)	—	13 (16.25%)	4 (14.29%)
Hyperlipidemia, *n* (%)	—	7 (8.75%)	3 (10.71%)
Coronary artery disease, *n* (%)	—	7 (8.75%)	5 (17.86%)
CVD score, *n* (%)	—	42/17/11/9/1/0	14/6/4/3/1/0
Biomarker			
NT-pro BNP, pg/mL	—	154.0 (49.0, 455.0)	256.0 (49.0,743.0)^†^
Troponin T, pg/mL	—	21.00 (7.1, 59.4)	41.33 (18.7, 99.1)^†^
Cancer treatment			
Anthracyclines, *n* (%)	—	14 (17.50%)	2 (7.14%)
Targeted agents, *n* (%)	—	24 (30.00%)	19 (67.86%)^†^
HER2-targeted therapies, *n* (%)		13 (16.25%)	7 (25.00%)
EGFR-TKI, *n* (%)		18 (22.50%)	8 (28.57%)
Anti-VEGF, *n* (%)		12 (15.00%)	5 (17.86%)
ICIs, *n* (%)	—	26 (32.50%)	9 (32.14%)
Antimetabolic, *n* (%)	—	23 (28.75%)	8 (28.57%)
Antimicrotubule agents, *n* (%)	—	36 (45.00%)	7 (25.00%)
Alkylating agents, *n* (%)	—	62 (77.50%)	15 (53.57%)^†^
Platinum analogues		54 (67.50%)	11 (39.29%)^†^
Cyclophosphamide		13 (20.97%)	4 (14.29%)
Chest radiotherapy, *n* (%)	—	17 (21.25%)	7 (25.00%)
Number of therapy cycles, No.	—	3.0 (2.0,6.0)	4.0 (2.0,6.0)
Time to CMR, m		6.25 (2.54,16.89)	4.02 (1.88,13.48)
Type of cancer			
Lung cancer, *n* (%)	—	32 (40.00%)	13 (46.43%)
Breast cancer	—	15 (18.75%)	3 (10.71%)
Digestive system cancer, *n* (%)	—	12 (15.00%)	6 (21.43%)
Hematologic malignancy, *n* (%)	—	7 (8.75%)	3 (10.71%)
Other cancers, *n* (%)	—	14 (17.50%)	3 (10.71%)
Medications, *n* (%)			
ACE inhibitors/ARBs		9 (11.25%)	6 (21.43%)
Beta-blockerse		18 (22.50%)	7 (25.00%)
Diuretic		16 (20.00%)	7 (25.00%)
Statinse		14 (17.50%)	6 (21.43%)

Data given as the mean ± SD, *n* (%), or median (25th to 75th percentile). BMI, body mass index; BP, blood pressure; CV, cardiovascular; CVD, cardiovascular disease; NT-pro BNP, N-terminal pro–B-type natriuretic peptide; EGFR-TKI, epidermal growth factor receptor tyrosine kinase inhibitor; Anti-VEGF, anti-vascular endothelial growth factor; ICIs, immune checkpoint inhibitors.

* *p* < 0.05 vs. controls.

† *p* < 0.05 vs. patients without LGE.

Pre-existing coronary heart disease was present in 11.11% of the patients with cancer. However, there were no significant differences in coronary artery disease, other cardiovascular risk factors, or cardiovascular disease score between the two groups. We observed that NT-pro BNP and troponin T were significantly higher in the LGE+ group than in the LGE− group [256.0 (49.0,743.0) vs. 154.0 (49.0, 455.0); 41.33 (18.7, 99.1) vs. 21.00 (7.1, 59.4); respectively; *p* < 0.05]. There was no significant difference in the cancer type, medications or whether it was combined with metastasis among the two subgroups. The proportion of patients with a therapeutic regimen that included targeted therapy was significantly higher in the LGE+ group than in the LGE− group (67.86% vs. 30.00%). However, there was no significant difference in time to CMR or number of therapy cycles between the LGE+ and the LGE− patients.

### CMR imaging results

**Figure 2:**
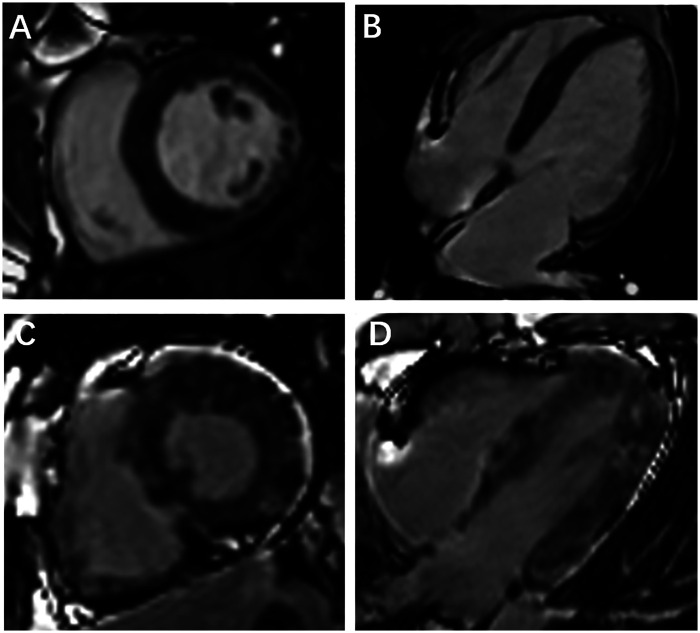
Representative images of LGE+ patients with cancer: multiple LGE predominantly subepicardial patterns **(A,B)**; extensive LGE with predominantly midmyocardial and subepicardial patterns **(C,D)**.

**Figure 3:**
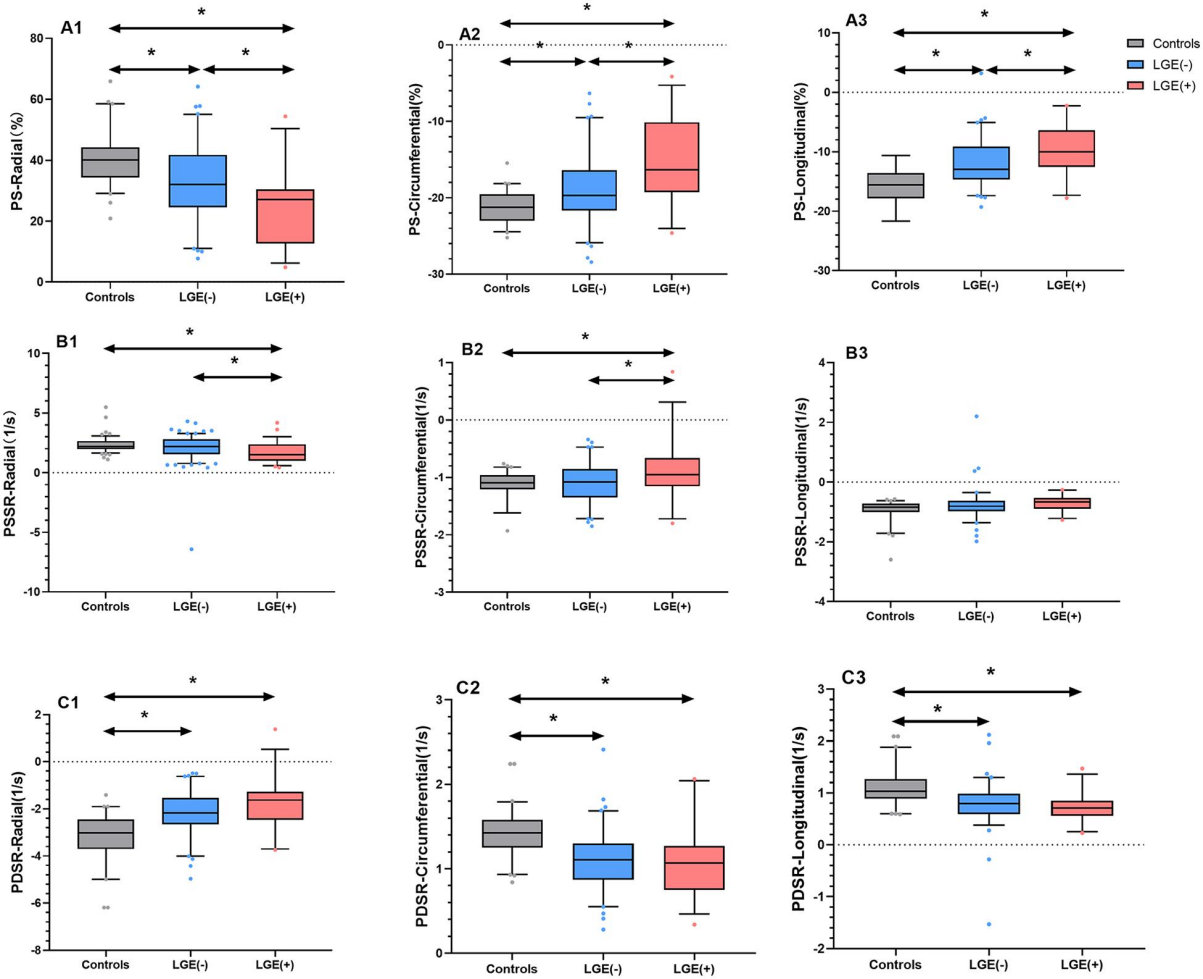
Differences in LV global, radial, circumferential, and longitudinal PS **(A1–A3)**, PSSR **(B1–B3)**, and PDSR **(C1–C3)** among the healthy controls, patients with cancer who were LGE−, and patients with cancer who were with LGE+, *p < 0.05.

Our results showed that 28 (25.93%) patients with cancer exhibited abnormal LGE, with the percentage of LGE was 6.79 (3.09, 12.33) (LGE%, 5SD). On CMR images, LGE mainly occurred at midmyocardium and/or subepicardial of the LV (23/28, 82.14%) (Figure 2). Table 2 presents the data on the functional and strain parameters of the LV of the control subjects, LGE− group, and LGE+ group. The LVEF values were lower in the LGE+ and LGE− groups than in the healthy controls (all *p* < 0.05), and LVEF was significantly lower in the LGE+ group than in the LGE− (*p* < 0.05). The prevalence of LV systolic dysfunction (LVEF < 55%) was significantly higher in the LGE+ group than in the LGE− group (60.71% vs. 23.75%, *p* < 0.05). Compared with the normal controls, the LV radial, circumferential and longitudinal PS of patients with LGE and those without LGE significantly impaired (all *p* < 0.05). The LV radial, circumferential, and longitudinal PS were significantly lower in the LGE+ group than in the LGE− group (all *p* < 0.05). Besides, the LV longitudinal, circumferential, and radial PDSR also were significantly lower in both patient groups than in the controls (all *p* < 0.05). Only the LV radial and circumferential PSSR were significantly lower in the LGE+ group than in the LGE− group and control subjects (all *p* < 0.05) (Figure 3).

**Table 2:** CMR parameters between normal individuals, cancer patients without or with LGE.

CMR measurements	Normal *n* = 60	Patients (LGE−) *n* = 80	Patients (LGE+) *n* = 28
LVEDVI, mL/m^2^	72.10 (64.82, 79.44)	70.86 (57.45, 82.51)	69.39 (60.22,78.99)
LVESVI, mL/m^2^	24.22 (19.99, 28.14)	24.26 (20.03, 32.77)	27.50 (23.70, 36.67)
LVSVI, mL/m^2^	46.18 (41.27, 52.14)	41.71 (34.25, 47.99)*	36.86 (27.35, 47.51)*
LVEF, %	65.85 (62.61, 70.15)	62.62 (56.34, 67.94)*	53.43 (46.86, 64.20)^*^^,^^†^
PS (%)			
Radial	40.11 (34.32, 44.17)	32.11 (24.51, 41.74)*	27.11 (12.71, 30.48)^*^^,^^†^
Circumferential	−21.27(−23.00, −19.55)	−19.70(−21.66, −16.40)*	−16.35(−19.26, −10.13)^*^^,^^†^
Longitudinal	−15.57(−17.85, −13.59)	−13.00(−14.66, −9.15)*	−10.02(−12.57, −6.41)^*^^,^^†^
PSSR (1/s)			
Radial	2.18 (1.97, 2.67)	2.20 (1.54, 2.82)	1.51 (1.00, 2.38)^*^^,^^†^
Circumferential	−1.10(−1.21, −0.96)	−1.08(−1.35, −0.86)	−0.96(−1.16, −0.66)^*^^,^^†^
Longitudinal	−0.83(−0.98, −0.71)	−0.81(−0.98, −0.63)	−0.67(−0.89, −0.53)
PDSR (1/s)			
Radial	−3.01(−3.71, −2.34)	−2.17(−2.66, −1.53)*	−1.63(−2.46, −1.27)*
Circumferential	1.43 (1.25, 1.58)	1.11 (0.87, 1.30)*	1.07 (0.75, 1.27)*
Longitudinal	1.03 (0.89, 1.27)	0.80 (0.59, 0.99)*	0.71 (0.56, 0.85)*
LVEF <55% (*n*, %)		19 (23.75%)	17 (60.71%)^†^
LEG% (5SD)		—	6.79(3.09, 12.33)

Data given as the median (25th, 75th percentile). LV, left ventricular; end diastolic volume; ESV, end systolic volume; SV, stroke volume; EF, ejection fraction; I, indexed to BSA; PS, Peak Strain; PSSR, Peak Systolic Strain Rate; PDSR, Peak Diastolic Strain Rate.

* *p* < 0.05 vs. controls.

† *p* < 0.05 vs. patients without LGE.

### Correlation between the clinical data and CMR parameters

**Figure 4:**
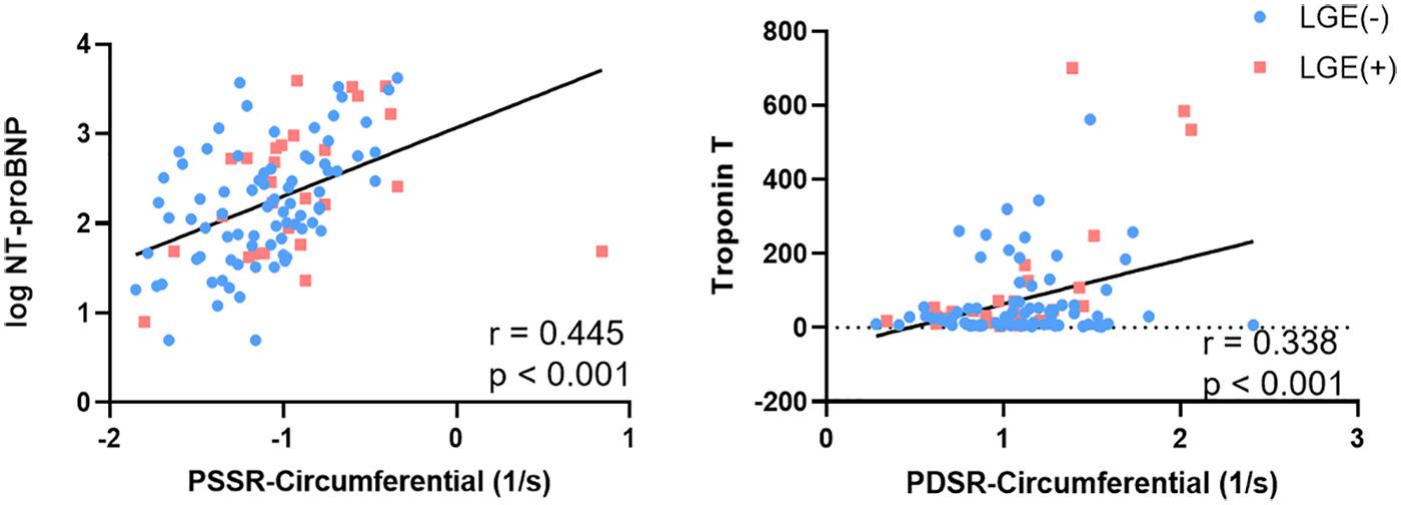
Correlation of NT-pro BNP (was log-transformed) with the circumferential PSSR **(A)**, and troponin T with the circumferential PDSR **(B)** in cancer patients.

**Figure 5:**
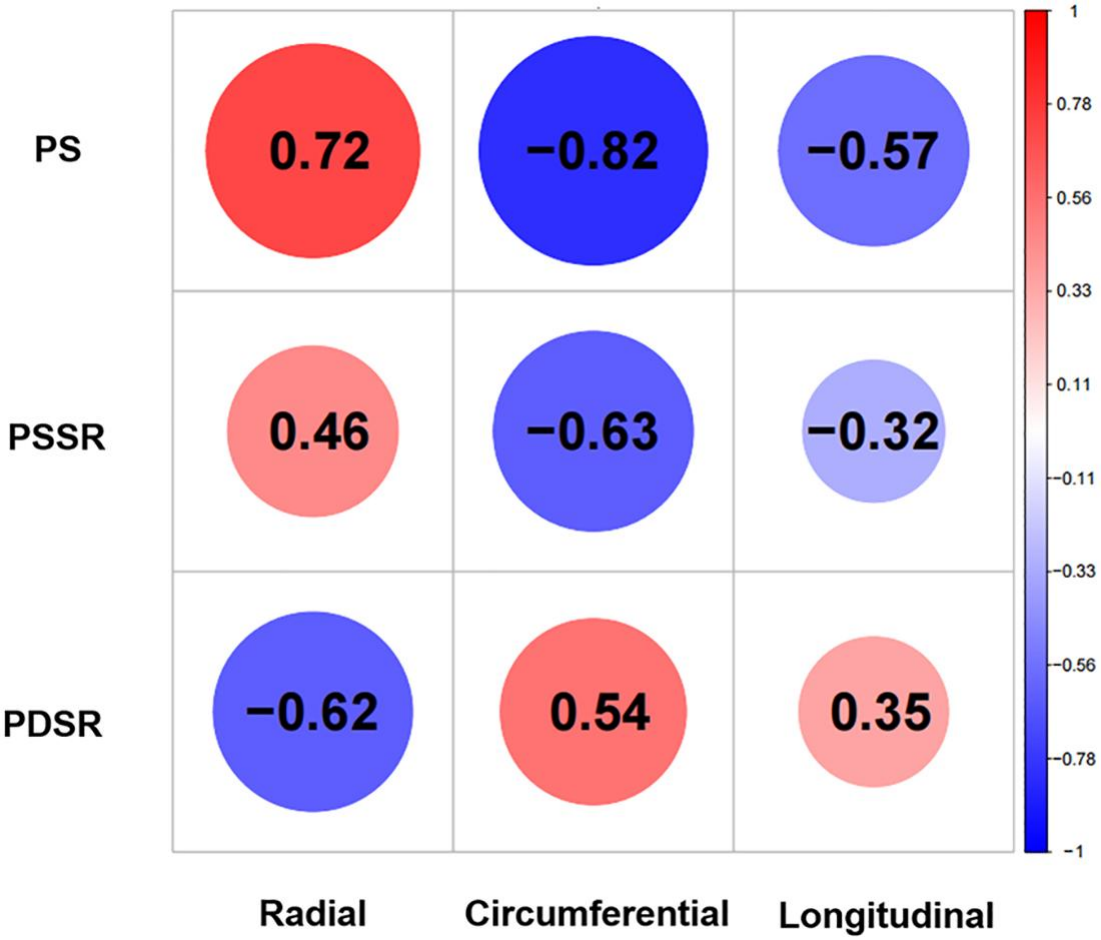
Heatmap shows relationship between LV global strain parameters in all directions and LVEF.

In the multivariate analysis the factors that could potentially influence LV strain, including age, sex, BMI, systolic blood pressure, cardiovascular risk factors, cardiac biomarker, therapeutic regimens, and the LGE extent were adjusted. Multivariate linear regression analysis revealed that NT-pro BNP was independently associated with the radial, circumferential and longitudinal PS (*β* = −0.419, *β* = 0.407, *β* = 0.327, respectively; *p* < 0.001) (Table 3). The NT-pro BNP was independently associated with the circumferential PSSR (*β* = 0.362, *p* < 0.001) (Supplementary Table S1). In addition, the troponin T was independently associated with the circumferential PDSR (*β* = 0.342, *p* < 0.001) (Supplementary Table S2) (Figure 4). The LGE extent was independently associated with circumferential PS (*β* = 0.297, *p* < 0.001). No significant correlations between cardiovascular risk factors and LV strain parameters were found. Besides, there was no statistically significant correlation between the cancer type, time to CMR, number of therapy cycles and the LGE extent in cancer patients.Pearson correlation analysis was used to examine the correlations between LV strain parameters and LVEF (Figure 5). There were moderate to high correlations between circumferential PDSR, PSSR, PS, and LVEF in the patients with cancer (*r* = 0.54, *r* = −0.63, *r* = −0.82, respectively; *p* < 0.001). The radial PDSR, PSSR, and PS were weak to moderately associated with LVEF (*r* = −0.62, *r* = 0.46, *r* = 0.72, respectively; *p* < 0.001). While, weak to moderate correlations between longitudinal PDSR, PSSR, PS, and LVEF in the patients with cancer were found (*r* = 0.35, *r* = −0.32, *r* = −0.57, respectively; *p* < 0.001).

**Table 3:** Multivariate linear regression of factors associated with left ventricle peak strain .

Variables	Radial PS				Circumferential PS				Longitudinal PS			
	Univariable		Multivariable		Univariable		Multivariable		Univariable		Multivariable	
	*r*	*p*	*β*	*p*	*r*	*p*	*β*	*p*	*r*	*p*	*β*	*p*
Age	0.640	0.510			−0.009	0.926			−0.031	0.754		
Male gender	−0.073	0.453			0.067	0.493			−0.027	0.778		
BMI	0.026	0.791			−0.093	0.338			0.024	0.804		
Systolic BP	0.128	0.186			0.200	0.038	−0.207	0.008	−0.012	0.898		
LGE%(5SD)	−0.282	0.003	−0.187	0.030	0.410	<0.001	0.297	<0.001	0.304	0.001	0.237	0.010
NT-pro BNP^&^	−0.459	<0.001	−0.419	<0.001	0.500	<0.001	0.407	<0.001	0.383	<0.001	0.327	<0.001
Troponin T	0.175	0.070	−0.138	0.101	−0.189	0.050	−0.122	0.132	−0.111	0.252		
Smoking	0.083	0.393			−0.126	0.192			−0.083	0.395		
Hypertension	−0.054	0.582			0.019	0.846			0.095	0.330		
Diabetes	0.218	0.023	0.231	0.006	−0.208	0.031	−0.136	0.080	−0.142	0.143		
Hyperlipidemia	−0.002	0.983			0.037	0.701			0.033	0.746		
Prior coronary artery disease	−0.071	0.466			0.054	0.580			−0.008	0.938		
Anthracyclines	−0.129	0.183			0.098	0.314			0.024	0.808		
Targeted agents	−0.265	0.006	−0.145	0.089	0.262	0.006	0.127	0.120	0.208	0.031	0.100	0.295
Immune checkpoint inhibitor	0.072	0.457			−0.088	0.367			−0.113	0.246		
Antimetabolic	0.036	0.708			−0.062	0.521			0.083	0.391		
Antimicrotubule agents	0.074	0.466			−0.123	0.203			−0.032	0.739		
Alkylating agents	0.144	0.137			−0.227	0.018	−0.166	0.035	−0.012	0.898		
Chest radiotherapy	0.073	0.454			−0.084	0.386			−0.045	0.646		

Factors with *p* < 0.1 in the univariable analysis were included in the multivariable analysis.

& NT-proBNP is log-transformed before being included in the regression analysis.

## Discussion

The current clinical diagnosis of cardiotoxicity is mainly based on the variable cutoff values of LVEF in different imaging modalities (8–10). However, LVEF may not be sufficiently sensitive to detect early myocardial injury, and reductions may reflect the systemic changes associated with cardiotoxicity after significant damage. More than half of patients do not fully recover from depressed LVEF after treatment for cardiotoxicity evaluated by LVEF (11, 12). Early detection of cardiotoxicity and timely initiation of cardioprotective therapy may help reverse its course. Reportedly, early cardiotoxicity is histologically characterized by myocardial edema and inflammation (13, 14), and eventually myocardial and extracellular fibrosis (15), both of which can be manifested as LGE. Previous study had demonstrated that the presence of LGE may reflect irreversible myocardial injury, and that patients with LGE also had a significantly increased chance of subsequent cardiac events (16).

In the current study, although there were no significant differences in the cardiovascular risk factors between the LGE− and LGE+ cohorts, the prevalence of LGE was relatively high (25.93%). Our study did not exclude patients at higher risk for cardiovascular factors, which provides greater generalizability in real-world routine clinical practice. In patients treated with anthracyclines and/or trastuzumab, LGE was present in only a minority of patients (17, 18). Unlike those in previous studies, our subjects were physician referred patients with cancer for CMR primarily to assess suspected cardiotoxicity. While, the presence of LGE can be as high as 42% to 74% in patients with confirmed ICI-associated myocarditis and ibrutinib-associated cardiotoxic, especially with a mid-myocardial pattern (19–21). We speculated that the variation in the prevalence of LGE reflected the cohort of patients with cancer who were referred for CMR, LGE is a characteristic manifestation of myocardial injury caused by cancer treatment. Our data also showed that LGE was highest in the patients with a therapeutic regimen that included targeted therapy. Trastuzumab is a typical targeted therapy that causes type II cardiotoxicity; it is rarely used alone and is often applied sequentially or concurrently as adjuvant cancer therapy, which may increase susceptibility to myocardial injury (8). In this study, LVEF was significantly lower in the patients with LGE than in those without. Therefore, we recommend LGE as an adjunct diagnostic indicator in addition to LVEF for diagnosing tumor treatment-related cardiotoxicity and as a clinical decision-making aid.

Our results showed that the LV global strain parameters of patients with cancer decreased to different degrees, even in patients without LGE, indicating that strain abnormalities precede detectable fibrosis and therefore serve as an earlier marker of myocardial injury. The global longitudinal strain (GLS) has been recommended by cardio-oncology guidelines for imaging evaluation of cardiotoxicity, defining a relative GLS decline of >15% during cancer treatment as cancer therapy–related cardiac dysfunction (CTRCD) (22). However, among the correlations between the strain parameters and LVEF in patients with cancer, the highest were for the circumferential strain parameters, indicating that circumferential strain reduction is probably the predominant mechanism related to LV dysfunction. Furthermore, the circumferential strain is the most reliable and reproducible measure of myocardial deformation (23). A previous study showed that changes in global circumferential strain could help predict declines in LVEF for at least 2 years after treatment in cancer survivors (24). Narayan et al. also found that global circumferential strain was the strongest predictor of cardiotoxicity (25). Overall, our results suggest that global circumferential strain (GCS) is a more reliable indicator than GLS for cardiotoxicity.

In this study, the radial, circumferential and longitudinal PS were significantly lower in the LGE+ group than in the LGE− group, while the LGE extent was independently associated with circumferential PS. This is consistent with the LGE distribution that reflects myocardial injury in the current study, which mainly occurred at the midmyocardium and/or subepicardial of the LV. The fibers in the midmyocardium were circumferentially oriented, which implies that the midmyocardium was responsible for the circumferential strain (26, 27). In addition, contraction of subepicardial muscle fibers also contribute to circumferential shortening of the myocardium (26). These results provide further evidence that the circumferential contraction of myocardium might be the most depressed in patients with cancer who received cardiotoxic therapy.

In addition to imaging, elevation of cardiac biomarkers can help with recognition of cardiotoxicity. An elevated natriuretic peptide level often represents hemodynamic congestion, while an elevated troponin is a marker of myocardial injury (28). Our study revealed that NT-pro BNP was independently associated with circumferential PSSR, consistent with previous research showing NT-pro BNP as a biomarker for preclinical systolic dysfunction (12). Besides, the troponin T was independently associated with circumferential PDSR, suggesting that troponin T may be a biomarker for diastolic dysfunction. Previous studies have shown that elevated troponin levels appeared to identify patients who did not recover from LVEF after chemotherapy (29). These findings further support the importance of monitoring NT-pro BNP and troponin T levels in patients receiving cancer treatment. Diagnostic accuracy could be improved by combining multiple imaging and cardiac biomarkers. This should contribute to early detection of cancer therapy-related cardiotoxicity.

Identifying the presence and severity of cardiotoxicity is essential in determining whether a patient can safely resume cancer therapy, thus profoundly influencing future oncological treatment decisions. Clinically, cardiotoxicity defined by reduced LVEF in tumor therapy could miss the onset of tissue-level myocardial changes and subclinical dysfunction. This study used CMR found that the combination of LGE, circumferential strains, and biomarkers could help clinicians diagnose myocardial injury that truly represents cardiotoxicity. However, CMR cannot be used as a routine examine to monitor cancer therapy-related myocardial injury in clinical practice due to accessibility and affordability. We recommend that CMR be performed in patients with a high clinical suspicion of cardiotoxicity rather than in all cancer patients. Developing a practical clinical score based on cardiac imaging and biomarkers to determine the cancer therapy-cardiotoxicity risk will help clinicians make decisions regarding the surveillance frequency and indication for cardio-protection. However, further long-term follow up of this study's cohort is needed to establish this score.

Our study has several limitations. First, this was a retrospective study involving a relatively small cohort of patients referred for CMR because of clinical suspicion of cardiotoxicity, rather than all cancer patients, which may have introduced selection bias. Nonetheless, the aim of this study was to provide a real-world perspective on routine clinical practice. This patient sample also explains the higher prevalence of LGE in this study than in other reported studies. Second, we did not determine the presence and extent of cardiotoxicity by myocardial biopsy; however, because of its invasiveness, potential for sampling error, and risk of serious complications, biopsy is now rarely performed. Third, long-term follow up data are not yet available; therefore, the precise prognostic significance of LGE and LV strain changes remains to be determined. Future studies are required to determine if these patients with LGE experience higher rates of cardiovascular events.

## Conclusions

The present study investigated the role of CMR for diagnosis of cancer patients with suspected cardiotoxicity in routine clinical practice. This study revealed that the presence of LGE was independently associated with circumferential PS in patients received oncotherapy, and the circumferential strain reduction is the predominant mechanism of LV dysfunction. LGE and circumferential strains could help clinicians diagnose myocardial injury that indicates cancer therapy-cardiotoxicity, which will help clinicians make decisions regarding the indication for cardio-protection.

## Data Availability

The original contributions presented in the study are included in the article/Supplementary Material, further inquiries can be directed to the corresponding authors.
