# Supplementary material for: Late gadolinium enhancement CMR for detecting myocardial injury after cancer therapy: a real-world observational study
Source: Front Cardiovasc Med. 2026 May 1;13:1787820. doi: 10.3389/fcvm.2026.1787820 (PMC13175857; doi:10.3389/fcvm.2026.1787820)
Supplement: Supplementary file 1 [file Datasheet1.docx]

**Table S1. Multivariate linear regression of factors associated with left ventricle peak systolic strain rate**

|  | **Radial PSSR** | | | |  | **Circumferential PSSR** | | | |  | **Longitudinal PSSR** | | | |
| --- | --- | --- | --- | --- | --- | --- | --- | --- | --- | --- | --- | --- | --- | --- |
|  | **Univariable** | | **Multivariable** | |  | **Univariable** | | **Multivariable** | |  | **Univariable** | | **Multivariable** | |
|  | r | p | β | p |  | r | p | β | p |  | r | p | β | p |
| Age |  |  |  |  |  |  |  |  |  |  |  |  |  |  |
| Male gender | -0.135 | 0.081 | 0.169 | 0.061 |  | 0.138 | 0.075 | 0.054 | 0.506 |  | 0.052 | 0.503 | 0.130 | 0.164 |
| BMI | 0.026 | 0.734 |  |  |  | 0.081 | 0.295 |  |  |  | 0.064 | 0.408 |  |  |
| Systolic BP | 0.117 | 0.132 |  |  |  | -0.115 | 0.137 |  |  |  | -0.095 | 0.221 |  |  |
| LGE%(5SD) | -0.123 | 0.204 |  |  |  | 0.340 | <0.001 | 0.186 | 0.022 |  | 0.081 | 0.407 |  |  |
| NT-pro BNP ^&^ | -0.262 | 0.006 | -0.245 | 0.008 |  | 0.445 | <0.001 | 0.362 | <0.001 |  | 0.238 | 0.014 | 0.236 | 0.013 |
| Troponin T | 0.176 | 0.068 | 0.113 | 0.226 |  | -0.228 | 0.018 | -0.219 | 0.05 |  | -0.126 | 0.195 |  |  |
| Smoking | 0.063 | 0.517 |  |  |  | 0.751 | 0.108 |  |  |  | 0.832 | 0.108 |  |  |
| Hypertension | 0.004 | 0.963 |  |  |  | -0.070 | 0.474 |  |  |  | -0.004 | 0.969 |  |  |
| Diabetes | 0.192 | 0.046 | 0.154 | 0.088 |  | -0.143 | 0.141 |  |  |  | -0.170 | 0.078 | -0.138 | 0.140 |
| Hyperlipidemia | 0.029 | 0.764 |  |  |  | -0.096 | 0.324 |  |  |  | -0.013 | 0.895 |  |  |
| Prior coronary artery disease | -0.171 | 0.077 | -0.054 | 0.567 |  | 0.153 | 0.115 |  |  |  | 0.107 | 0.269 |  |  |
| Anthracyclines | -0.199 | 0.039 | -0.233 | 0.011 |  | 0.168 | 0.081 | 0.082 | 0.394 |  | 0.104 | 0.286 |  |  |
| Targeted agents | -0.205 | 0.034 | -0.095 | 0.318 |  | 0.225 | 0.019 | 0.099 | 0.227 |  | 0.153 | 0.115 |  |  |
| Immune checkpoint inhibitor | 0.241 | 0.012 | 0.191 | 0.036 |  | -0.202 | 0.019 | -0.145 | 0.065 |  | -0.190 | 0.048 | -0.206 | 0.029 |
| Antimetabolic | 0.108 | 0.267 |  |  |  | -0.204 | 0.035 | -0.130 | 0.110 |  | -0.010 | 0.917 |  |  |
| Antimicrotubule agents | 0.035 | 0.720 |  |  |  | -0.101 | 0.298 |  |  |  | -0.018 | 0.854 |  |  |
| Alkylating agents | 0.222 | 0.021 | 0.131 | 0.153 |  | -0.371 | <0.001 | -0.313 | <0.001 |  | -0.138 | 0.154 |  |  |
| Chest radiotherapy | 0.030 | 0.755 |  |  |  | 0.020 | 0.834 |  |  |  | -0.075 | 0.440 |  |  |

**Table S2. Multivariate linear regression of factors associated with left ventricle peak diastolic strain rate**

|  | **Radial PDSR** | | | |  | **Circumferential PDSR** | | | |  | **Longitudinal PDSR** | | | |
| --- | --- | --- | --- | --- | --- | --- | --- | --- | --- | --- | --- | --- | --- | --- |
|  | **Univariable** | | **Multivariable** | |  | **Univariable** | | **Multivariable** | |  | **Univariable** | | **Multivariable** | |
|  | r | p | β | p |  | r | p | β | p |  | r | p | β | p |
| Age | 0.044 | 0.572 |  |  |  | -0.205 | 0.008 | -0.114 | 0.209 |  | -0.180 | 0.020 | -0.173 | 0.023 |
| Male gender | -0.188 | 0.015 | -0.063 | 0.491 |  | 0.164 | 0.033 | 0.127 | 0.148 |  | 0.210 | 0.006 | 0.180 | 0.018 |
| BMI | 0.049 | 0.531 |  |  |  | 0.039 | 0.612 |  |  |  | -0.003 | 0.971 |  |  |
| Systolic BP | -0.152 | 0.049 | -0.085 | 0.369 |  | 0.138 | 0.075 |  |  |  | 0.091 | 0.240 |  |  |
| LGE%(5SD) | 0.320 | <.001 | 0.204 | 0.030 |  | -0.222 | 0.021 | -0.110 | 0.220 |  | 0.014 | 0.884 |  |  |
| NT-pro BNP ^&^ | 0.289 | 0.003 | 0.234 | 0.011 |  | -0.310 | 0.001 | -0.293 | <0.001 |  | -0.107 | 0.274 |  |  |
| Troponin T | -0.198 | 0.04 | -0.203 | 0.022 |  | 0.338 | <0.001 | 0.342 | <0.001 |  | 0.158 | 0.103 |  |  |
| Smoking | 0.134 | 0.166 |  |  |  | 0.042 | 0.666 |  |  |  | -0.027 | 0.782 |  |  |
| Hypertension | 0.054 | 0.58 |  |  |  | 0.042 | 0.670 |  |  |  | 0.042 | 0.667 |  |  |
| Diabetes | -0.164 | 0.090 | -0.073 | 0.420 |  | 0.170 | 0.078 | 0.061 | 0.488 |  | 0.085 | 0.380 |  |  |
| Hyperlipidemia | 0.047 | 0.632 |  |  |  | -0.044 | 0.651 |  |  |  | -0.008 | 0.937 |  |  |
| Prior coronary artery disease | 0.153 | 0.114 |  |  |  | -0.046 | 0.638 |  |  |  | -0.017 | 0.857 |  |  |
| Anthracyclines | 0.112 | 0.250 |  |  |  | -0.142 | 0.142 |  |  |  | 0.036 | 0.712 |  |  |
| Targeted agents | 0.313 | <0.001 | 0.210 | 0.021 |  | -0.187 | 0.053 | -0.105 | 0.241 |  | -0.146 | 0.131 |  |  |
| Immune checkpoint inhibitor | -0.099 | 0.306 |  |  |  | 0.089 | 0.362 |  |  |  | 0.12 | 0.215 |  |  |
| Antimetabolic | -0.08 | 0.408 |  |  |  | 0.064 | 0.508 |  |  |  | -0.053 | 0.589 |  |  |
| Antimicrotubule agents | -0.037 | 0.701 |  |  |  | 0.069 | 0.479 |  |  |  | 0.043 | 0.656 |  |  |
| Alkylating agents | -0.144 | 0.136 |  |  |  | 0.223 | 0.021 | 0.179 | 0.041 |  | 0.041 | 0.671 |  |  |
| Chest radiotherapy | -0.018 | 0.852 |  |  |  | 0.100 | 0.305 |  |  |  | 0.096 | 0.324 |  |  |
